# Cardiovascular Health, Adiposity, and Food Insecurity in an Underserved Population

**DOI:** 10.3390/nu11061376

**Published:** 2019-06-19

**Authors:** Candice A. Myers, Corby K. Martin, Robert L. Newton, John W. Apolzan, Connie L. Arnold, Terry C. Davis, Eboni G. Price-Haywood, Peter T. Katzmarzyk

**Affiliations:** 1 Pennington Biomedical Research Center, 6400 Perkins Road, Baton Rouge, LA 70808, USA; corby.martin@pbrc.edu (C.K.M.); robert.newton@pbrc.edu (R.L.N.J); john.apolzan@pbrc.edu (J.W.A.); peter.katzmarzyk@pbrc.edu (P.T.K.); 2 Department of Medicine, Feist-Weiller Cancer Center, Louisiana State University Health Sciences Center, 1501 Kings Highway, Shreveport, LA 71103, USA; carnol@lsuhsc.edu (C.L.A.); tdavis1@lsuhsc.edu (T.C.D.); 3 Ochsner Clinic Foundation, Center for Outcomes and Health Services Research, 1514 Jefferson Highway, New Orleans, LA 70121, USA; eboni.pricehaywood@ochsner.org; 4 Ochsner Clinical School, University of Queensland, 1401 Jefferson Highway, New Orleans, LA 70121, USA

**Keywords:** cardiovascular health, adiposity, food insecurity, health literacy, underserved population

## Abstract

This study investigated associations between cardiovascular health (CVH), adiposity, and food insecurity by race, sex, and health literacy in a sample of 800 underserved patients with obesity (body mass index [BMI] ≥ 30 kg/m^2^). CVH was assessed using American Heart Association Life’s Simple 7 (LS7) and adiposity was estimated using BMI and waist circumference (WC). Mixed models including interaction terms between food insecurity and sex, race, and health literacy were analyzed for LS7, BMI, and WC. Stratified models were analyzed as indicated by significant interactions. Mean BMI and WC were 37.3 kg/m^2^ (4.6 SD) and 113.5 cm (12.4 SD), respectively. Among patients, 31% were food insecure and 31% had low health literacy. There were significant positive associations between food insecurity and BMI (*p* = 0.03) and WC (*p* = 0.03) in the overall sample. In sex-stratified models, women who were food insecure had higher BMI (*p* = 0.02) and WC (*p* = 0.007) than their food secure counterparts. Further, food insecure patients with better health literacy had greater BMI (*p* = 0.004) and WC (*p* = 0.007) than their food secure counterparts. Results suggest that adiposity is a greater burden in food insecure patients, which may be an important consideration for obesity treatment in underserved populations.

## 1. Introduction

Cardiovascular disease remains a leading cause of mortality in the United States (US) [1] with obesity being an important risk factor [2,3]. Both cardiovascular disease and obesity are interconnected, non-communicable diseases that are particularly burdensome among underserved and minority populations, including noted sex and race differences [4,5,6,7,8]. Efforts to better understand disparities in cardiovascular health and obesity have highlighted the role of social determinants. For example, a recent American Heart Association (AHA) scientific statement reinforces the important influence of social determinants such as socioeconomic position and race on cardiovascular disease risk [8].

Food insecurity has recently emerged as an important social determinant of health as evidence demonstrating its relationship with adverse health outcomes and health disparities continues to accumulate. Food insecurity is defined as the lack of “nutritionally adequate and safe foods” or the limited or uncertain “ability to acquire acceptable foods in socially acceptable ways” [9]. Food insecurity has been shown to be more prevalent in women [10] and racial minorities [11]. Importantly, food insecurity has been broadly linked to chronic disease [12,13,14] with a wealth of studies showing a strong association between food insecurity and obesity [15,16,17,18]. There is also evidence that food insecurity is related to poor cardiovascular health [19], as measured using the AHA Life’s Simple 7 (LS7) [20], and increased cardiovascular disease risk [12,21,22].

Cardiovascular disease research has also pointed to the importance of health literacy in understanding disease risks and outcomes [23,24,25]. Defined by the U.S. Department of Health and Human Services (HHS) and the National Academy of Medicine (NAM), health literacy is the “degree to which individuals have the capacity to obtain, process, and understand health information and services needed to make health decisions” [26,27]. Failure by providers and healthcare systems to account for deficits in these capacities may contribute to poor health outcomes. The AHA’s scientific statement addressing health literacy and cardiovascular disease calls for better integration of health literacy into management and prevention strategies targeting cardiovascular disease, especially given noted disparities in health literacy by sex and race [28]. Such calls to action are particularly relevant as studies have shown that low health literacy is associated with increased risk of cardiovascular disease [25], while high health literacy has been linked to lower body weight [29].

Given the aforementioned evidence, there is a need to recognize the interplay of multiple social determinants and health disparities in the relationship between food insecurity and health outcomes. The aim of the current study is to examine potential differences in the associations between food insecurity and cardiovascular health and measures of adiposity by sex, race, and health literacy status. This objective was carried out in a large sample of underserved adults with obesity.

## 2. Materials and Methods

### 2.1. Participants

Data for this study are from the Promoting Successful Weight Loss in Primary Care in Louisiana (PROPEL) trial (ClinicalTrials.gov Identifier NCT02561221). PROPEL is a two-year cluster-randomized, two-arm controlled trial conducted in 18 primary care clinics across Louisiana to test the effectiveness of a pragmatic, high intensity, lifestyle-based obesity treatment program in an underserved population. All data reported here are from baseline assessments collected 2016-2017. Patient characteristics and data collection procedures for PROPEL have been discussed elsewhere [30]. Briefly, patient recruitment occurred in primary care clinics via identification of potential patients using multiple approaches, including interactions with their primary care providers, searches of electronic medical records, responses to emails sent through their health care provider health portal, responses to study recruitment materials (e.g., posters, brochures) available in clinic waiting areas, and interactions with PROPEL staff in the clinic. The Pennington Biomedical Research Center Institutional Review Board approved the study protocol and all patients provided written informed consent. Study technicians who did not deliver the intervention conducted all assessments, including anthropometric and blood pressure measurements, fasting glucose and lipids, concomitant medications, and questionnaires.

### 2.2. Dependent Variables

Cardiovascular health was measured using LS7 [20]. LS7 is a composite measure of seven cardiovascular health behaviors and factors, including smoking, diet, physical activity, BMI, blood pressure, cholesterol, and glucose. Each LS7 component is scored as ideal (2 points), intermediate (1 point), or poor (0 points) based upon predefined criteria [20]. Table 1 presents the scoring metric for each LS7 component. Based on scoring criteria for each component, a LS7 total score was calculated as a composite cardiovascular health score with a potential range of 0–14, with lower scores indicating poorer cardiovascular health. As done previously [31], the LS7 total score was further categorized as ideal (scores 11–14 points), intermediate (scores 9–10), and poor (scores 0–8). 

Smoking status was assessed via a self-reported questionnaire. The original published criteria for the diet score included five components capturing fruits and vegetables, fish, fiber-rich whole grains, sodium, and sugar-sweetened beverages [20]. However, the dietary intake questionnaire utilized in PROPEL focused on dietary fat, fruit, vegetable, and alcohol intake [30]. Given this, a healthy diet score was created that measured three dietary intake components, including fruits (≥1 time/day), vegetables (≥1 time/day), and fat (low in fat during past 12 months). Physical activity was measured using the International Physical Activity Questionnaire — Short Form (IPAQ-SF) [32]. Resting systolic and diastolic blood pressures were obtained using an automated Omron device (HEM-907XL). Fasting finger-stick blood samples were obtained, and total cholesterol and glucose were assayed as previously described [30]. Concomitant medications reported at baseline were incorporated into the scoring metrics for blood pressure, total cholesterol, and glucose. Within the PROPEL patient sample, 56%, 28%, and 25% reported taking anti-hypertensive, glucose-lowering, and lipid-lowering medications, respectively.

Two measures were used to assess adiposity: Body mass index (BMI) and waist circumference (WC). Height and weight were measured in duplicate using a portable stadiometer (Seca Model 213) and digital scale (Seca Model 876), respectively, and BMI was calculated (weight (kg) / height (m^2^)). Waist circumference was measured in duplicate using an inelastic anthropometric tape at the mid-point between the lower rib and the iliac crest. If the two measurements differed by >0.5 cm, 0.5 kg, and 0.5 cm for height, weight, and waist circumference, respectively, a third measurement was obtained and the two closest measurements were averaged for analysis.

### 2.3. Independent Variable

Food security status was measured using the 6-Item Food Security Survey Module, which is a well-validated questionnaire that has been used in studies assessing health literacy [33,34]. This questionnaire references food availability over the past 12 months. Total scores ranged from 0–6, with higher scores indicating greater food insecurity. Two or more affirmative answers indicated food insecurity.

### 2.4. Covariates

Health literacy was assessed using the Rapid Estimate of Adult Literacy in Medicine short form (REALM-SF), which includes seven health-related words that patients read aloud [35]. A score of six or less indicated reading comprehension below high school level (low health literacy), while a score of seven indicated reading comprehension at the high school level or greater.

Sex, race (White, African American, Other [American Indian or Alaska Native, Asian, Hawaiian/Pacific Islander, Multi-race, Other]) age, education, income, and marital status were self-reported via a demographic questionnaire.

### 2.5. Statistical Analysis

Bivariate analyses to test for significant differences in all measures between food security groups included independent samples t-tests for continuous variables and chi-square tests for categorical variables. The objective of the statistical analyses was to assess a priori differences in the relationships between food insecurity and cardiovascular health and adiposity by sex, race, and health literacy status. This was achieved via linear mixed effects models with interaction terms between food insecurity and sex, race, and health literacy for each of the three dependent variables: LS7 total score, BMI, and WC. Stratified models were then analyzed as indicated by significant interaction terms. To further assess the association between cardiovascular health and food insecurity, the LS7 total score and each component were treated as binary outcomes using logistic mixed effects models. This was achieved by combining ideal and intermediate categories into a single indicator (= 1) with poor (= 0) as the reference. All models included age, education, income, and marital status as covariates, were adjusted for multiple comparisons (Tukey-Kramer), and accounted for clustering of patients across clinics (SAS version 9.4, PROC MIXED and PROC GLIMMIX). The final analytic sample consisted of 800 PROPEL patients with BMI ≥ 30.0 kg/m^2^ at baseline.

## 3. Results

For all PROPEL patients, the mean LS7 total score was 6.7 (1.9 SD; 0–11 range) and mean BMI and WC were 37.3 kg/m^2^ (4.6 SD; 30.0–50.0 range) and 113.5 cm (12.4 SD; 81.0–158.0 range), respectively. Thirty one percent of patients reported being food insecure and 31% had low health literacy. Almost 85% of patients were female and over 67% of patients were African American. The mean age of patients was 49.4 years (13.1 SD) and 4% reported being Hispanic.

Patient characteristics and LS7 components are reported stratified by food security status in Table 2 and Table 3, respectively. The mean LS7 total score was not significantly different by food security status. BMI (*p* = 0.002) and WC (*p* = 0.01) were significantly higher in food insecure patients compared to food secure patients. There were no significant differences in the percent of female patients between food secure and food insecure groups. There were significantly more African American patients who reported being food insecure compared to food secure (*p* = 0.01). Health literacy scores were significantly lower in food insecure patients (*p*= 0.01), with significantly more food insecure patients having poorer health literacy (*p* < 0.001). We also report patient characteristics and LS7 components stratified by sex and race in Tables S1 and S2.

**Figure 1:**
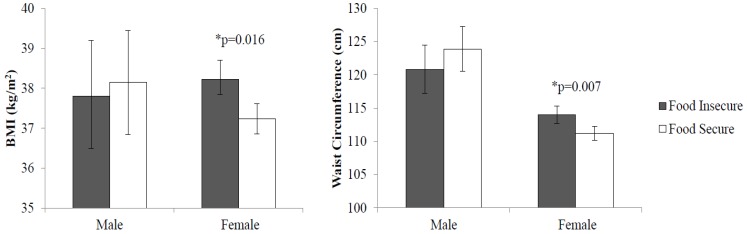
Adjusted Mean Adiposity by Food Security Status and Sex in PROPEL Patients. * indicates statistical significance (*p* < 0.05). BMI, body mass index.

Food insecurity was significantly and positively associated with BMI (*p* = 0.03) and WC (*p* = 0.03) in the overall sample. There was a significant food insecurity by sex interaction for WC (*p* = 0.02), but not for BMI (*p* = 0.23). However, given the significant correlation established between BMI and WC, [36] as well as that found in the PROPEL sample (Pearson’s r = 0.73, *p* < 0.001), sex-stratified models were assessed for both adiposity measures. Figure 1 shows that women who were food insecure had greater BMI (38.2 compared to 37.2 kg/m^2^; *p* = 0.02) and WC (114.0 compared to 111.2 cm; *p* = 0.007) than their food secure counterparts. Associations between adiposity and food insecurity were not significant in men. Results did not indicate that food insecurity and cardiovascular health were significantly related or any significant food insecurity by sex differences in LS7 scores.

**Figure 2:**
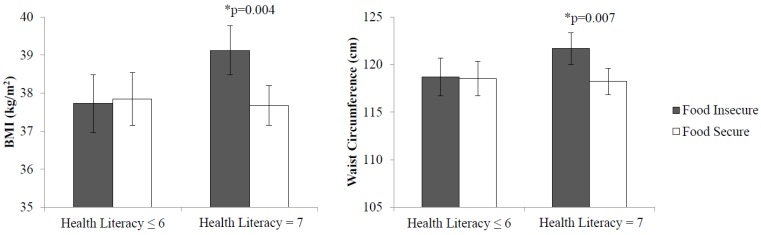
Adjusted Mean Adiposity by Food Security Status and Health Literacy Status in PROPEL Patients. * indicates statistical significance (*p* < 0.05). BMI, body mass index.

Food insecurity by health literacy interactions were significant for both BMI (*p* = 0.03) and WC (*p* = 0.04). As shown in Figure 2, health literacy-stratified models showed that food insecure patients with better health literacy had greater BMI (39.1 compared to 37.7 kg/m^2^; *p* = 0.004) and WC (121.7 compared to 118.2 cm; *p* = 0.01) than their food secure counterparts. However, these associations were not significant in those with low health literacy. No health literacy-specific differences were shown in the relationship between LS7 scores and food insecurity.

Results did not indicate any food insecurity by race interactions for LS7, BMI, or WC.

Table 4 presents adjusted odds ratios and 95% confidence intervals from logistic mixed models testing the association between ideal/intermediate cardiovascular health and food insecurity. This included the LS7 total score and each of the seven components. No significant associations were demonstrated between the LS7 total score or individual components and food insecurity. We also assessed blood pressure, cholesterol, and glucose as continuous measures using mixed models and found no indication of significant associations between these cardiovascular health factors and food insecurity (data not shown). Further, there were no food insecurity by sex, race, or health literacy interactions for the LS7 total score or its components (dichotomous or continuous).

## 4. Discussion

Using baseline data from a large cluster-randomized controlled trial in an underserved population with obesity, this study examined associations between food insecurity and cardiovascular health and adiposity. Importantly, this study explicitly focused on potential differences in these associations by noted disparities, including sex, race, and health literacy status. Results indicated a number of significant differences, specifically in the relationship between food insecurity and adiposity. This included greater BMI and WC in food insecure women compared to women who reported being food secure. These findings are particularly relevant given a recent meta-analysis that found a robust positive association between food insecurity and obesity, with this relationship being most significant in adult women [16], as well as a smaller study that found a significant association between food insecurity and WC in a sample of low-income minority women [37].

Adiposity was further shown to be significantly greater in food insecure patients with better health literacy compared to patients who reported being food secure. This finding is interesting as research has largely shown a significant link between low health literacy and greater body weight in adults [29,38]. While similar differences in adiposity were not demonstrated between food insecure and food secure patients with low health literacy, this finding does highlight the significant and pervasive link between food insecurity and obesity [15,16]. Further, while better health literacy may support better health decisions and, in turn, improved health outcomes, better health literacy in the context of food insecurity may not be actionable, so that food insecurity maintains its deleterious health consequences. Importantly, to the authors’ knowledge, this study was the first to examine the interaction between health literacy and food insecurity in relation to cardiovascular health and adiposity. Given this, more studies that examine the interconnected influence of food insecurity and health literacy on health outcomes are warranted, especially in other populations with different sociodemographic and health profiles.

The results did not demonstrate any significant associations between food insecurity and cardiovascular health metrics. Other studies that have found significant linkages between food insecurity and cardiovascular health were most often carried out in large, nationally representative samples and did not rely upon LS7 to measure cardiovascular health [12,21]. Only one other study has investigated food insecurity in relation to LS7 and it components [19]. This study found that being food insecure was significantly linked to a decreased likelihood of ‘good’ cardiovascular health (ideal and intermediate levels of LS7 total score combined) [19]. This study also found that, contrary to hypothesized expectations, those who reported food insecurity were significantly more likely to have ideal levels of blood pressure and total cholesterol [19]. Importantly, this study used data from a population-based representative sample of Wisconsin residents and a single question to assess food security status [19]. Further, their sample was predominantly White (85%) [19], while the PROPEL trial has greater racial diversity with a majority of African American patients (67%). Such study-dependent complexities point to the fact that empirical evidence regarding food insecurity and cardiovascular health, LS7 in particular, is only beginning to accumulate and further investigations are needed to better explicate this relationship. The use of similar standardized measures of food insecurity and cardiovascular health would further aid in comparing and summarizing results from studies in this field.

No significant race by food insecurity interactions were found in the associations with cardiovascular health or adiposity. This is potentially due to the lack of significant differences between racial groups in cardiovascular health and adiposity measures within the sample (see Tables S1 and S2). Further, the study sample is largely African American, which is a particularly unique attribute of this trial. While food insecurity was significantly disparate between racial groups, the lack of variation in the selected outcomes (i.e., a majority of patients had poor cardiovascular health) may have limited the ability to detect any significant race-based differences. This is not to say that racial disparities in food insecurity, cardiovascular health, and adiposity are not important, but rather in this sample, sex and health literacy emerged as the more relevant social determinants when considering how food insecurity is linked to health outcomes. Further investigations in additional samples are warranted to better elucidate the complex interplay of multiple social determinants in shaping how food insecurity impacts various health outcomes.

This study has a number of key strengths. First, most data for the outcomes assessed in this study were derived from clinic- and laboratory-based assessments, with the exception of smoking, diet, and physical activity, which were collected via self-report questionnaires. Second, the PROPEL trial is being carried out in an underserved and largely minority (i.e., African American) population, which makes the results from the current study broadly generalizable to similar populations across the US [30]. Further, this trial is also being conducted in Louisiana, which has a significantly higher food insecurity rate (17.3%) than the U.S. overall (11.8%), but is comparable to other Southern states, including Arkansas, Mississippi, and Alabama, where food insecurity is also notably high [39]. This makes the current study particularly relevant within a state and region and that has a demonstrated need to better understand how food insecurity, as a prevalent health-related disparity, contributes to poor health. Results from this study certainly address this public health need.

Importantly, a few limitations are noted for this study. First, this was a cross-sectional investigation, which limits the ability to address causality between food insecurity and cardiovascular health and adiposity. Further, the purpose of the PROPEL trial is to test the effectiveness of an obesity treatment program, which potentially creates a self-selection bias among patients who are seeking such treatment and qualify to participate in the trial. Subsequently, all PROPEL patients have obesity, which potentially limits variation in cardiovascular health and associated metrics as this sample may be largely unhealthier than samples observed in other studies or the general population. Third, while the REALM is one of the most widely used validated instruments to assess health literacy and the short form version reduces participant burden, some researchers have critiqued this tool as only an assessment of an individual’s ability to read and pronounce health-related words rather than accurately reflecting an individual’s level of health literacy [40]. Last, the interaction terms and subsequent stratified analyses assessed in this study create disparate patient numbers between subgroups (e.g., sex, race, health literacy). Smaller sample sizes within certain subgroups may potentially drive the significant or non-significant effects shown in this study.

## 5. Conclusions

In conclusion, significant differences in adiposity were seen in food insecure compared to food secure women, as well as in food insecure compared to food secure patients with better health literacy. These data suggest that adiposity is a greater burden in food insecure patients and may pose challenges for obesity treatment in underserved populations. That is, patients seeking weight loss who also report being food insecure may start a treatment program with greater adiposity compared to food secure patients. Further, the state of food insecurity may serve as a hurdle to successful weight loss if not considered as a relevant factor in the delivery of an intervention. Such considerations are important not only for overall effectiveness of an intervention, but also ensuring equity in intervention response among patients with heterogeneous adiposity at baseline and differing social determinants.

## Figures and Tables

**Table 1:** Definitions of Ideal, Intermediate, and Poor Levels of Life’s Simple 7 (LS7) Components.

Component	Ideal (2 Points)	Intermediate (1 Point)	Poor (0 Points)
Smoking ^a^	Never or quit > 12 months	Former ≤ 12 months	Current
Healthy diet ^a,b^	3 components	1 to 2 components	0 components
Physical activity ^a^	≥150 min/week moderate intensity or ≥75 min/week vigorous intensity or ≥150 min/week moderate + vigorous	1–149 min/week moderate intensity or 1–74 min/week vigorous intensity or 1–149 min/week moderate + vigorous	None
BMI	<25 kg/m^2^	25 to 29.9 kg/m^2^	≥30 kg/m^2^
Blood pressure	<120/<80 mmHg untreated	SBP 120 to 139 mm Hg or DBP 80 to 89 mm Hg or treated to ideal level	SBP ≥ 140 or DBP ≥ 90 mm Hg
Total cholesterol	<200 mg/dL untreated	200 to 239 mg/dL or treated to ideal level	≥240 mg/dL
Glucose	<100 mg/dL untreated	100 to 125 mg/dL or treated to ideal level	≥126 mg/dL

^a^ Based on self-report questionnaires; all other measures based on physical or laboratory values. ^b^ Measures three dietary intake components: (1) Fruits: ≥1 time/day; (2) vegetables: ≥ 1 time/day; (3) fat: Diet low in fat (previous 12 months). Ideal score (all three components) captures a diet with greater fruit and vegetable intake and low in fat. BMI, body mass index; SBP, systolic blood pressure; DBP, diastolic blood pressure; LS7, Life’s Simple 7.

**Table 2:** Baseline Characteristics of Promoting Successful Weight Loss in Primary Care in Louisiana (PROPEL) Patients by Food Security Status.

	Food Secure (*n* = 554)	Food Insecure (*n* = 246)	*p*-Value
BMI (kg/m^2^)	37.0 (4.5)	38.0 (4.8)	**0.002**
Waist circumference (cm)	112.7 (12.6)	115.4 (12.0)	**0.005**
Systolic blood pressure (mmHg)	123.4 (16.0)	121.8 (17.3)	0.211
Diastolic blood pressure (mmHg)	79.2 (10.2)	79.0 (11.4)	0.745
Total cholesterol (mg/dL)	179.6 (37.3)	180.0 (37.0)	0.875
Glucose (mg/dL)	107.3 (32.6)	113.2 (42.1)	**0.034**
Female	460 (83.0)	217 (88.2)	0.061
African American	359 (64.8)	179 (72.8)	**0.005**
Health literacy	6.5 (1.1)	6.3 (1.2)	**0.013**
≤6 (≤8^th^ grade)	150 (27.1)	97 (39.4)	**<0.001**
7 (≥9^th^ grade)	404 (72.9)	149 (60.6)	
Age	50.0 (13.4)	48.2 (12.4)	0.078
Education			**<0.001**
Less than HS	39 (7.0)	22 (8.9)	
HS	107 (19.3)	71 (28.9)	
Some college	216 (39.0)	118 (48.0)	
Bachelor’s degree	109 (19.7)	23 (9.3)	
Postgraduate degree	82 (14.8)	12 (4.9)	
Income (annual family)			**<0.001**
< $10,000	74 (13.4)	82 (33.3)	
$10,000-$19,999	105 (19.0)	63 (25.6)	
$20,000-$39,999	124 (22.4)	66 (26.8)	
$40,000-$59,999	94 (17.0)	23 (9.3)	
≥$60,000	143 (25.8)	9 (3.7)	
Marital status			**<0.001**
Married	237 (42.8)	62 (25.2)	
Divorced/separated	123 (22.2)	88 (35.8)	
Never married	148 (26.7)	81 (32.9)	
Widowed	46 (8.3)	15 (6.1)	

Continuous variables are reported as mean standard deviation (SD). Categorical variables are reported as n (%). Boldface indicates statistical significance (*p* < 0.05). BMI, body mass index; HS, high school.

**Table 3:** LS7 Scores for PROPEL Patients by Food Security Status.

	Food Secure (*n* = 554)	Food Insecure (*n* = 246)	*p*-Value
LS7	6.8 (1.8)	6.5 (1.9)	0.073
Poor	423 (76.4)	191 (77.6)	0.405
Intermediate	94 (17.0)	36 (14.6)	
Ideal	7 (1.3)	1 (0.4)	
Smoking			**0.012**
Poor	51 (9.2)	34 (13.8)	
Intermediate	15 (2.7)	14 (5.7)	
Ideal	488 (88.1)	197 (80.1)	
Healthy diet score			0.418
Poor	378 (68.2)	178 (72.4)	
Intermediate	165 (29.8)	66 (26.8)	
Ideal	9 (1.6)	2 (0.8)	
Physical activity			0.643
Poor	256 (46.2)	108 (43.9)	
Intermediate	73 (13.2)	38 (15.4)	
Ideal	217 (39.2)	93 (37.8)	
BMI			----
Poor	554 (100.0)	146 (100.0)	
Intermediate	0.0 (0)	0.0 (0)	
Ideal	0.0 (0)	0.0 (0)	
Blood pressure			0.933
Poor	124 (22.4)	54 (22.0)	
Intermediate	314 (56.7)	138 (56.1)	
Ideal	115 (20.8)	54 (22.0)	
Total cholesterol			0.372
Poor	36 (6.5)	19 (7.7)	
Intermediate	231 (41.7)	89 (36.2)	
Ideal	269 (48.6)	127 (51.6)	
Glucose			0.077
Poor	90 (16.2)	50 (20.3)	
Intermediate	178 (32.1)	88 (35.8)	
Ideal	274 (49.5)	100 (40.7)	

Continuous variables are reported as mean (SD). Categorical variables are reported as n (%). Boldface indicates statistical significance (*p* < 0.05). LS7, Life’s Simple 7.

**Table 4:** Adjusted Odds Ratios (AORs)^a^ and 95% Confidence Intervals (CI) of LS7 and Components by Food Insecurity.

LS7^b^	Food Insecurity
Total score	0.97 (0.61, 1.55)
Smoking	0.92 (0.55, 1.53)
Healthy diet	0.82 (0.57, 1.18)
Physical activity	1.33 (0.95, 1.88)
BMI	----
Blood pressure	1.08 (0.72, 1.61)
Total cholesterol	0.80 (0.42, 1.51)
Glucose	0.74 (0.48, 1.14)

^a^ AORs were adjusted for food insecurity, health literacy, race, sex, age, income, education, and marital status. ^b^ Intermediate and ideal categories were combined (= 1); reference category is poor (= 0). LS7, Life’s Simple 7.

## Supplementary Materials

The following are available online at https://www.mdpi.com/2072-6643/11/6/1376/s1, Table S1: Baseline Characteristics of PROPEL Patients by Sex and Race, Table S2: LS7 Scores for PROPEL Patients by Sex and Race.
