# Supplementary material for: Cardiovascular Health, Adiposity, and Food Insecurity in an Underserved Population
Source: Nutrients. 2019 Jun 19;11(6):1376. doi: 10.3390/nu11061376 (PMC6628173; doi:10.3390/nu11061376)
Supplement: Supplementary file 1 [file nutrients-11-01376-s001.pdf]

Table S1. Baseline Characteristics of PROPEL Patients by Sex and Race.

	Male (n=123)	Female (n=677)	*p*-value	White (n=207)	African American (n=538)	Other (n=55)	*p*-value
BMI (kg/m^2)	37.4 (4.5)	37.3 (4.7)	0.699	37.3 (4.6)	37.2 (4.6)	38.2 (5.0)	0.325
Waist circumference (cm)	122.7 (11.9)	111.8 (11.8)	**<0.001**	114.6 (13.3)	113.0 (12.3)	114.2 (10.6)	0.419
Systolic blood pressure (mmHg)	127.9 (16.8)	122.0 (16.2)	**<0.001**	120.0 (15.7)	124.1 (16.2)	121.6 (19.1)	**0.003**
Diastolic blood pressure (mmHg)	80.0 (10.7)	79.0 (10.6)	0.321	78.1 (9.8)	79.6 (10.5)	78.9 (13.8)	0.348
Total cholesterol (mg/dL)	169.4 (40.4)	181.6 (36.3)	**0.001**	181.4 (37.9)	178.3 (37.1)	187.0 (34.5)	0.157
Glucose (mg/dL)	120.3 (40.3)	107.1 (34.7)	**<0.001**	110.9 (37.5)	108.6 (35.7)	107.6 (31.1)	0.457
Food security [0-6]	0.9 (1.7)	1.4 (2.0)	**0.010**	1.0 (1.8)	1.5 (2.1)	1.4 (1.9)	**0.002**
Food secure	76.4 (94)	67.9 (460)	0.061	77.3 (160)	66.7 (359)	63.6 (65)	**0.013**
Food insecure	23.6 (29)	32.1 (217)		22.7 (47)	33.3 (179)	36.4 (20)	
Female	----	----		74.9 (155)	88.5 (476)	83.6 (46)	**<0.001**
African American	50.4 (62)	70.3 (476)	**<0.001**	----	----	----	
Health literacy [0-7]	6.3 (1.2)	6.4 (1.2)	0.388	6.7 (0.7)	6.3 (1.2)	6.3 (1.5)	**<0.001**
≤6 (≤8th grade)	35.8 (44)	30.0 (203)	0.201	17.9 (37)	35.7 (192)	32.7 (18)	**<0.001**
7 (≥9th grade)	64.2 (79)	70.0 (474)		82.1 (170)	64.3 (346)	67.3 (37)	
Age	54.0 (11.4)	48.6 (13.2)	**<0.001**	50.3 (13.8)	49.5 (12.7)	45.3 (13.5)	**0.028**
Education			**0.001**				0.053
Less than HS	7.3 (9)	7.7 (52)		8.2 (17)	6.7 (36)	14.5 (8)	
HS	15.4 (19)	23.5 (159)		25.6 (53)	21.0 (113)	21.8 (12)	
Some college	33.3 (41)	43.3 (293)		33.3 (69)	45.7 (246)	34.5 (19)	
Bachelor's degree	28.5 (35)	14.3 (97)		20.8 (43)	14.7 (79)	18.2 (10)	
Postgraduate degree	15.5 (19)	11.1 (75)		12.1 (25)	11.7 (63)	10.9 (6)	
Income (annual family)			**<0.001**				**<0.001**
< $10,000	11.4 (14)	21.0 (142)		10.6 (22)	22.1 (119)	27.3 (15)	
$10,000-$19,999	10.6 (13)	22.9 (155)		19.3 (40)	21.6 (116)	21.8 (12)	
$20,000-$39,999	16.3 (20)	25.1 (170)		20.8 (43)	25.1 (135)	21.8 (12)	
$40,000-$59,999	17.9 (22)	14.0 (95)		12.6 (26)	15.8 (85)	10.9 (6)	
≥$60,000	39.8 (49)	15.2 (103)		33.8 (70)	13.6 (73)	16.4 (9)	
Marital status			**<0.001**				**0.019**
Married	55.3 (68)	34.1 (231)		47.3 (98)	34.0 (183)	32.7 (18)	
Divorced/separated	19.5 (24)	27.6 (187)		23.7 (49)	27.9 (150)	21.8 (12)	

Never married	23.6 (29)	29.5 (200)		24.2 (50)	29.4 (158)	38.2 (21)	
Widowed	1.6 (2)	8.7 (59)		4.8 (10)	8.7 (47)	7.3 (4)	

Continuous variables are reported as mean (SD). Categorical variables are reported as n (%). Statistically significant differences were assessed using independent samples t-tests for continuous variables and chi-square tests for categorical variables. Boldface indicates statistical significance ($p < 0.05$).

Table S2. LS7 Scores for PROPEL Patients by Sex and Race.

	**Male (n=123)**	**Female (n=677)**	***p-value***	**White (n=207)**	**African American (n=538)**	**Other (n=55)**	***p-value***
LS7 [0-14]	6.3 (1.7)	6.8 (1.9)	**0.016**	6.6 (1.9)	6.8 (1.9)	6.7 (1.6)	0.386
Poor	83.7 (103)	75.5 (511)	0.094	76.8 (159)	76.2 (140)	81.8 (45)	0.794
Intermediate	9.8 (12)	17.4 (118)		17.4 (36)	16.4 (88)	10.9 (6)	
Ideal	0.8 (1)	1.0 (7)		1.0 (2)	0.9 (5)	1.8 (1)	
Smoking			0.407				**0.013**
Poor	7.3 (9)	11.2 (76)		15.5 (32)	8.0 (43)	18.2 (10)	
Intermediate	3.3 (4)	3.7 (25)		3.9 (8)	3.5 (19)	3.6 (2)	
Ideal	89.4 (101)	84.9 (575)		80.2 (166)	88.5 (476)	78.2 (43)	
Healthy diet			0.111				0.154
Poor	76.0 (94)	68.2 (462)		68.6 (142)	70.4 (379)	63.6 (35)	
Intermediate	23.6 (29)	29.8 (202)		31.3 (65)	27.5 (148)	32.7 (18)	
Ideal	0.0 (0)	1.6 (11)		0.0 (0)	1.7 (9)	3.6 (2)	
Physical activity			**0.002**				0.829
Poor	33.3 (41)	47.7 (323)		43.0 (89)	46.5 (250)	45.5 (25)	
Intermediate	12.2 (15)	14.2 (96)		15.5 (32)	13.0 (70)	3.6 (2)	
Ideal	52.8 (65)	36.2 (245)		40.1 (83)	38.5 (207)	49.1 (27)	
BMI			----				----
Poor	100 (123)	100 (677)		100 (207)	100 (538)	100 (55)	
Intermediate	0.0 (0)	0.0 (0)		0.0 (0)	0.0 (0)	0.0 (0)	
Ideal	0.0 (0)	0.0 (0)		0.0 (0)	0.0 (0)	0.0 (0)	
Blood pressure			**<0.001**				0.498
Poor	28.5 (35)	21.1 (143)		19.3 (40)	23.8 (128)	18.2 (10)	
Intermediate	64.2 (79)	55.1 (373)		58.5 (121)	55.9 (301)	54.5 (30)	
Ideal	7.3 (9)	23.6 (160)		22.2 (46)	20.1 (108)	27.3 (15)	
Total cholesterol			**0.016**				**0.040**
Poor	7.3 (9)	6.8 (46)		7.2 (15)	6.5 (35)	9.1 (5)	

Intermediate	51.2 (63)	38.0 (257)		48.8 (101)	36.8 (198)	38.2 (21)	
Ideal	38.2 (47)	51.6 (349)		41.1 (85)	52.8 (284)	49.1 (27)	
Glucose			**<0.001**				0.956
Poor	30.9 (38)	15.1 (102)		18.4 (38)	16.9 (91)	20.0 (11)	
Intermediate	34.1 (42)	33.1 (224)		33.3 (69)	33.6 (181)	29.1 (16)	
Ideal	31.7 (39)	49.5 (335)		47.3 (98)	46.5 (250)	47.3 (26)	

Continuous variables are reported as mean (SD). Categorical variables are reported as n (%). Statistically significant differences were assessed using independent samples t-tests for continuous variables and chi-square tests for categorical variables. Boldface indicates statistical significance ($p < 0.05$).
